# Antioxidant, analgesic and anti-inflammatory activities of the methanolic extract of *Piper betle* leaves

**Published:** 2013

**Authors:** Badrul Alam, Fahima Akter, Nahida Parvin, Rashna Sharmin Pia, Sharmin Akter, Jesmin Chowdhury, Kazi Sifath-E-Jahan, Ekramul Haque

**Affiliations:** 1 *Department of Pharmacy, Atish Dipankar University of Science & Technology, Dhaka, Bangladesh*; 2 *Department of Pharmacy, University of Rajshahi, Rajshahi, Bangladesh*

**Keywords:** Analgesic, Antioxidant, Anti-inflammatory, *Piper betle*

## Abstract

**Objective:** The present study was designed to evaluate the antioxidant, analgesic, and anti-inflammatory activities of the methanolic extract of *Piper betle* leaves (MPBL).

**Materials and Methods:** MPBL was evaluated for anti-inflammatory activity using carrageenan-induced hind paw edema model. Analgesic activity of MPBL was evaluated by hot plate, writhing, and formalin tests. Total phenolic and flavonoids content, total antioxidant activity, scavenging of 1,1-diphenyl-2-picrylhydrazyl (DPPH) radical, peroxynitrate (ONOO) as well as inhibition of total ROS generation, and assessment of reducing power were used to evaluate antioxidant potential of MPBL.

**Results:** The extract of MPBL, at the dose of 100 and 200 mg/kg, produced a significant (p<0.05) increase in pain threshold in hot plate method whereas significantly (p<0.05) reduced the writhing caused by acetic acid and the number of licks induced by formalin in a dose-dependent manner. The same ranges of doses of MPBL caused significant (p<0.05) inhibition of carrageenan-induced paw edema after 4 h in a dose-dependent manner. In DPPH, ONOO^-^, and total ROS scavenging method, MPBL showed good antioxidant potentiality with the IC_50_ value of 16.33±1.02, 25.16±0.61 , and 41.72±0.48 µg/ml, respectively with a significant (p<0.05) good reducing power.

**Conclusion:** The findings of the study suggested that MPBL has strong analgesic, anti-inflammatory, and antioxidant effects, conforming the traditional use of this plant for inflammatory pain alleviation to its antioxidant potentiality.

## Introduction

Free radicals cause depletion of immune system antioxidants (Ouattara et al., 2011 ▶), change in gene expression and induce abnormal proteins, and contribute to more than one hundred disorders in humans including atherosclerosis, arthritis, ischemia, and reperfusion injury of many tissues, central nervous system injury, gastritis, cancer, and AIDS (Hela and Abdullah, 2010 ▶). Moreover, various free radicals are also responsible for the induction of short term algesia (Chung, 2004 ▶) as well as play an important role in the pathogenesis of inflammation (Winrow et al., 1993 ▶). Inflammation is the response to injury of cells and body tissues through different factors such as infections, chemicals, thermal, and mechanical injuries (Oyedapo et al., 2008 ▶). Various endogenous mediators such as histamine, serotonin, bradykinin, prostaglandins, etc. are most abundant in inflammatory cells and among them prostaglandins are ubiquitous substances that indicate and modulate cell and tissue responses involved in inflammation (Hossain et al., 2011 ▶). These mediators, even in small quantities, can elicit pain response. Pain results in dropped muscular activities, associated with various free radicals as well as reactive oxygen species (ROS) that trigger some second messengers and are involved in sensitization of dorsal horn neurons that plays a fundamentally important role in neuropathic pain (Ali and Salter, 2001 ▶; Zhang et al., 2003 ▶). Having various and severe adverse effects such as gastric lesions for NSAIDs, adverse cardiovascular thrombotic effects for selective cyclooxygenase-2 inhibitors (Chowdhury et al., 2009 ▶), and tolerance and dependence induced by opiates, use of these drugs as anti-inflammatory and analgesic agents have not been successful in all of the cases. Therefore, new anti-inflammatory and analgesic drugs lacking those effects are being searched all over the world as alternatives. Medicinal plant have great value to phytochemists because of their medicinal properties (Oladosu et al., 2011 ▶) so that, the study of plants that have been traditionally used as pain killers should still be seen as a fruitful and logical research strategy in the search for new analgesic drugs and pain mechanisms (Calixto et al., 2000 ▶). 

Betelvine *Piper betle* (*P. betle*) belongs to the family of Piperaceae, popularly regarded as a medicinal plant in the South East Asia region. Experimentally, leaves of *P. betle *are shown to possess antimicrobial (Agarwal et al., 2012 ▶), gastroprotective (Majumdar et al., 2003 ▶), wound healing (Santhanam and Nagarajan, 1990 ▶), hepatoprotective (Saravanan et al., 2002 ▶), antioxidant (Choudhary and Kale, 2002 ▶; Santhakumari et al., 2003 ▶), anti-fertility on male rats (Ratnasooriya and Premakumara, 1997 ▶), and antimotility effects on washed human spermatozoa (Ratnasooriya et al., 1990 ▶). The chief constituent of the leaves is a volatile oil which contains phenols, betel-phenol, chavibetol and chavicol, cadinene, and hydroxychavicol, which have been ascribed to possess anti-oxidant and anti-carcinogenic activities (Bhide et al., 1991 ▶; Garg and Jain, 1992 ▶; Singh et al., 2009 ▶). The tribal population and aborigines of Bangladesh chew the leaves as a narcotic which causes swooning and profuse sweating and also helps to give warmth to the body during winter. The present study was carried out to evaluate the antioxidant, analgesic, and anti-inflammatory activities of crude extract of *P. betle *leaves in different experimental model. 

## Materials and Methods

**Plant materials**

The leaves of the *P. betle *Linn were collected from the adjoining area of Jahangirnagar University Campus, Bangladesh, during February 2011. The plant material was taxonomically identified by the National Herbarium of Bangladesh and voucher specimen no. JU/33334 is maintained in our laboratory for future reference.

**Chemicals**

Ammonium molybdate, Folin-chiocaltu phenol reagent, and carrageenan were purchased from E. Merck (Germany). 1,1-diphenyl-2-picryl-hydrazyl (DPPH), ascorbic acid, quercetin potassium ferric cyanide, 2',7'-dichlorfluorescein-diacetate (DCFH-DA), 5,5’-dithiobis [2-nitrobenzoic acid] (DTNB), L-penicillamine (L-2-amino-3-mercapto-3-methylbutanoic acid), and diethylene triamine pentaacetic acid (DTPA) were purchased from Sigma Co. (St. Louis, MO, USA). 6-Hydroxy-2,5,7,8-tetramethylchroman-2-carboxylic acid (Trolox) was purchased from Aldrich Chemical Co. (Milwaukee, WI, USA) The high quality 2’,7’-dichlorofluorescein diacetate (DCFH-DA), dihydrorhoclamine 123 (DHR 123), and ONOO^-^ were purchased from Molecular Probes (Eugene, Oregon, USA) and Cayman (Ann Arbor, MI, USA), respectively. Nalbuphine, indomethacin, and diclofenac-Na were collected from Square Pharmaceuticals Ltd., Bangladesh. All other chemicals and reagents were of analytical grade.

**Preparation of plant extract**

The plant material was shade-dried with occasional shifting and then powdered with a mechanical grinder, passing through sieve #40, and stored in a tight container. The dried powder material (1.5 kg) was refluxed with MeOH for three hours. The total filtrate was concentrated to dryness, *in vacuo* at 40 ^° ^C to render the MeOH extract (410 g). 

***In vitro*** **antioxidant activity**

*The amount of phenolic compounds and flavonoids*

The total phenolic and flavonoid content of methanolic extract was determined using Folin-ciocalteu reagent (Yu et al., 2002 ▶) and aluminium chloride colorimetric method (Chang et al., 2002 ▶), respectively. The content of total phenolics in MPBL was calculated from regression equation of the calibration curve (y=0.013x+0.127, r^2^= 0.988) and is expressed as galic acid equivalents (GAE) and the flavonoid contents of the extract was expressed in terms of quercetin equivalent (the standard curve equation: y=0.009x-0.036).

*Determination of total antioxidant capacity*

The antioxidant activity of the MPBL was evaluated by the phosphomolybdenum method according to the procedure of Prieto et al., (1999) ▶. The assay is based on the reduction of Mo(VI)–Mo(V) by the extract and subsequent formation of a green phosphate/Mo(V) complex at acid pH. Extract (0.3 ml) was combined with 3 ml of reagent solution (0.6 M sulfuric acid, 28 mM sodium phosphate, and 4 mM ammonium molybdate). The tubes containing the reaction solution were incubated at 95 ^°^C for 90 min. Then, the absorbance of the solution was measured at 695 nm using a spectrophotometer (Shimadzu, UV-150-02) against blank after cooling to room temperature. Methanol (0.3 ml) is used as the blank experiment. The antioxidant activity is expressed as the number of equivalents of ascorbic acid using the following formula: 

C = (c×V)/m, where, C: total antioxidant activity, mg/g plant extract, in ascorbic acid; c: the concentration of ascorbic acid established from the calibration curve, mg/ml; V: the volume of extract, ml; m: the weight of pure plant extract, g.

*Free radical scavenging activity measured by DPPH*

The free radical scavenging activity of MPBL, based on the scavenging activity of the stable 1,1-diphenyl-2- picrylhydrazyl (DPPH) free radical, was determined by the method described by Braca et al. (2001) ▶. MPBL (0.1 ml) was added to 3 ml of a 0.004% MeOH solution of DPPH. Absorbance at 517 nm was determined after 30 min, and the percentage inhibition activity was calculated from [(A_0_–A_1_)/A_0_]×100, where A_0_ is the absorbance of the control and A_1 _is the absorbance of the extract/ standard. IC_50_ value was calculated from the equation of line obtained by plotting a graph of concentration (μg/ml) versus % inhibition.

*Measurement of the ONOO* ^-^ *scavenging activity*

The ONOO^-^ scavenging activity was measured by monitoring the oxidation of DHR 123, by modifying the method of Kooy et al. (1994) ▶. The DHR 123 (5 mM), in dimethylformamide, was purged with nitrogen, stored at -80 ^°^C and used as a stock solution. This solution was then placed on ice and kept from exposure to light, before the study. The buffer used consisted of 90 mM sodium chloride, 50 mM sodium phosphate, 5 mM potassium chloride at pH 7.4, and 100 mM diethylenetriaminopentaacetic acid (DTPA), each of which were prepared with high quality deionized water, and purged with nitrogen. The final concentration of the DHR 123 was 5 µM. The background and final fluorescent intensities were measured 5 minutes after treatment, both with and without the addition of authentic ONOO^-^. The DHR 123 was oxidized rapidly by authentic ONOO^-^, and its final fluorescent intensity remained unchanged over time. The fluorescent intensity of the oxidized DHR 123 was measured using a microplate fluorescence reader, FL 500 (Bio-Tek Instruments Inc.), with excitation and emission wavelengths of 480 and 530 nm, respectively. The results were expressed as the mean±standard error (n=3) of the final fluorescence intensity minus the background fluorescence. The effects were expressed as the percentage of inhibition of the DHR 123 oxidation. IC_50_ was calculated from the equation of line obtained by plotting a graph of concentration (μg/ml) versus % inhibition.

*Measurement of the inhibition of the total ROS generation*

Mice kidney homogenates, prepared from the kidneys of freshly killed male Swiss albino mice, weighing 30-39 g, were mixed with or without a suspension of extracts, and then incubated with 12.5 µM DCFH-DA, at 37 ^°^C for 30 min. Phosphate buffer (50 mM, pH 7.4) was used. DCFH-DA is a stable compound, which easily diffuses into cells, and is hydrolyzed by intracellular esterase to yield a reduced non-fluorescent compound, DCFH, which is trapped within the cells. The ROS produced by cells oxidize the DCFH to the highly fluorescent 2',7'-dichlorodihydrofluorescein (DCF). The fluorescence intensity of the oxidized DCF was monitored on a microplate fluorescence spectrophotometer (Bio-Tek Instruments Inc., Winooski, VT), with excitation and emission wavelengths of 460 and 530 nm, respectively (Label and Bondy, 1990 ▶). IC_50_ value was calculated from the equation of line obtained by plotting a graph of concentration versus % inhibition.

*Reducing power activity*

The reducing power of MPBL was determined according to the method previously described (Oyaizu, 1986 ▶). Extracts at different concentrations in 1 ml of 10% DMSO were mixed with 2.5 ml of phosphatebuffer (0.2 M, pH 6.6) and 2.5 ml potassium ferricyanide [K_3_Fe (CN)_6_] (1%), and then the mixture was incubated at 50 ^°^C for 30 min. Afterwards, 2.5 ml of trichloroacetic acid (10%) was added to the mixture, which was then centrifuged at 3000 rpm for 10 min. Finally, 2.5 ml of upper layer solution was mixed with 2.5 ml distilled water and 0.5 ml FeCl_3_ (0.1%), and the absorbance was measured at 700 nm. Increased absorbance of the reaction mixture indicated increased reducing power.

***In vivo*** **analgesic activity**

*Animal*

Swiss albino mice (25-30g) and Wistar rats (175-250 g) of both sexes were used for assessing biological activity. The animals were maintained under standard laboratory conditions and had free access to food and water *ad libitum. *The animals were allowed to acclimatize to the environment for 7 days prior to experimental session. The animals were divided into different groups, each consisting of five animals which were fasted overnight prior to the experiments. Experiments on animals were performed in accordance with guidelines of the Institutional Animal Ethics Committee, Atish Dipankar University of Science & Technology, Dhaka, Bangladesh. Animal treatment and maintenance for acute toxicity and analgesic effects were conducted in accordance with the Principle of Laboratory Animal Care (NIH publication No. 85-23, revised 1985) and the Animal Care and Use Guidelines of Atish Dipankar University of Science & Technology, Dhaka, Bangladesh.

*Acute toxicity study*

Acute oral toxicity assay was performed in healthy nulliparous and nonpregnant adult female albino Swiss mice (25-30 g) divided into different groups. The test was performed using increasing oral dose of the MPBL in water (50, 100, 200, 500, and 1000 mg/kg body weight) in 20 ml/kg volume to different test groups. Normal group received water. The mice were allowed to feed *ad libitum,* kept under regular observation for 48 h for any mortality or behavioral changes (Sanmugapriya and Venkataraman, 2006 ▶).

*Hot plate method*

The animals were divided into four groups with five mice in each group. Group I animals received vehicle (1% Tween 80 in water, 10 ml/kg body weight), animals of Group II received nalbuphine at 10 mg/kg body weight while animals of Group III and Group IV were treated with 100 and 200 mg/kg body weight (p.o.) of the MPBL*. *The animals were placed on Eddy’s hot plate kept at a temperature of (55±0.5) °C. A cut-off period of 15 second, was observed to avoid damage to the paw (Franzotti et al., 2000 ▶). Reaction time was recorded when animals licked their fore or hind paws, or jumped prior to 0, 30, 60, and 90 min after oral administration of the samples. 

*Acetic acid-induced writhing test*

The analgesic activity of the samples was also studied using acetic acid-induced writhing model in mice. Test samples and vehicle were administered orally 30 min before intraperitoneal administration of 0.7% v/v acetic acid but diclofenac-Na was administered intraperitonially 15 min before injection of acetic acid. After an interval of 5 min, the mice were observed for specific contraction of body referred to as ‘writhing’ for the next 10 min (Ahmed et al., 2004 ▶).

*Formalin test*

The antinociceptive activity of the drugs was determined using the formalin test described by Dubuission and Dennis (1977) ▶. Control group received 20 µl of 5% formalin via injection into the dorsal surface of the right hind paw 60 min after administration of MPBL (200 and 400 mg/kg, p.o.) and 30 min after administration of diclofenac Na (10 mg/kg, i.p.). The mice were observed for 30 min after the injection of formalin and the amount of time spent licking the injected hind paw was recorded. The first 5 min post-formalin injection is referred to as the early phase and the period between 15 and 30 min as the late phase. The total time spent licking or biting the injured paw (pain behavior) was measured with a stop watch. 

**Anti-inflammatory activity**

*Carrageenan-induced paw edema test in rats*

Wistar rats (175-250 g) of both sexes were divided into four groups of five animals each. The test groups received 100 and 200 mg/kg body weight (p.o.) of the extract. The reference group received indomethacin (10 mg/kg body weight, p.o.) while the control group received 3 ml/kg body weight normal saline. After 1 h, 0.1 ml of 1% carrageenan suspension in normal saline was injected into the subplanatar tissue of the right hind paw (Winter et al., 1962 ▶). The paw volume was measured at 1, 2, 3, and 4 h after carrageenan injection using a micrometer screw gauge. The percentage inhibition of the inflammation was calculated from the formula: 

% inhibition = (1-D_t/_D_o_)×100,

where, D_o_ was the average inflammation (hind paw edema) of the control group of rats at a given time and D_t_ was the average inflammation of the drug treated (i.e., extract or reference indomethacin) rats at the same time (Winter et al., 1962 ▶).

**Statistical analysis**

All values were expressed as the mean±SEM of three replicate experiments. The analysis was performed using SPSS statistical package for WINDOWS (version 16.0; SPSS Inc, Chicago). Results related to the reducing power activities were statistically analyzed by applying the Student t-test and p<0.001 were considered to be statistically significant. All in vivo data are subjected to ANOVA followed by Dunnett’s test and p<0.05 were considered to be statistically significant.

## Results

**Acute toxicity studies**

The acute toxicity studies mainly aim at establishing the therapeutic index, i.e., the ratio between the pharmacologically effective dose and the lethal dose on the same strain and species. MPBL was safe up to a dose of 1000 mg/kg (p.o.) body weight. Behavior of the animals was closely observed for the first 3 h then at an interval of every 4 h during the next 48 h. The extract did not cause mortality in mice and rats during 48 h observation but little behavioral changes, locomotor ataxia, diarrhea, and weight loss were observed. Food and water intake had no significant difference among the group studied. 

***In vitro*** **antioxidant activity**

*Total phenolic and flavonoid contents*

Table 1 represents the content of both groups in MPBL extract. The content of total phenolics in the extract of *P. betle* was determined using the Folin-ciocalteu assay, calculated from regression equation of the calibration curve (y=0.013x+0.127, r^2^= 0.988) and is expressed as galic acid equivalents (GAE) and the flavonoid contents of the extract was expressed in terms of quercetin equivalent (the standard curve equation: y=0.009x-0.036). 

*Total antioxidant capacity*

Total antioxidant capacity of MPBL is expressed as the number of equivalents of ascorbic acid (Table 1). Total antioxidant capacity of MPBL was found to be 81.72± 0.48 mg/gm equivalent of ascorbic acid. 

*DPPH radical scavenging activity*

The percentage (%) scavenging of DPPH radical was found to be concentration-dependent with the IC_50_ value of 16.33±0.16 µg/ml, while IC_50_ value of standard ascorbic acid was found to be 12.10±0.02 μg/ml (Table 2).

*Peroxynitrite (ONOO* ^-^ *) scavenging activity*

The ONOO^-^ scavenging activity was measured by monitoring the oxidation of DHR 123. The MeOH extract of MPBL exhibited significant ONOO^-^ scavenging effects in a dose-dependent manner, with IC_50_ values of 25.16±0.61μg/ml, whereas, penicillamine, a well-known ONOO^-^ scavenger, with IC_50_ value of 10.20±0.32 μg/ml. (Table 2). 

*Inhibition of total ROS generation*

The percentage inhibition of ROS generation was illustrated in Table 2 and it is observed that scavenging of ROS by the extract is also concentration-dependent with the IC_50_ value of 41.72±0.48 µg/ml, while IC_50_ value of standard trolox was found to be 12.32±0.11 μg/ml. 

*Reducing power ability*

**Figure 1:**
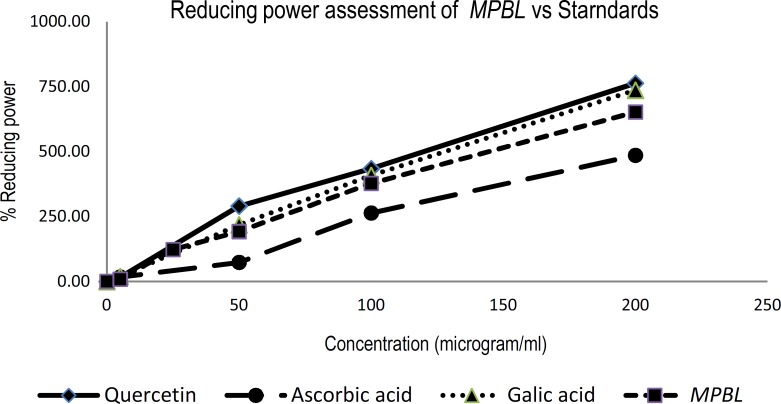
Values are mean±SEM, Reducing power of MPBL, quercetin, ascorbic acid, and galic acid by spectrophotometric detection of Fe^3^^+^ to Fe^2^^+^ transformation

For the measurement of the reductive ability, we investigated the Fe^3^^+^ to Fe^2^^+^ transformation in the presence of MPBL and compared with standards (Galic acid, quercetin and ascorbic acid) (Figure 1<

**Table 1:** Yield, total amount of plant phenolic compounds, flavonoids, and total antioxidant capacity of methanolic extract of *Piper betle*

**Sample**	**Yield (%)**	a **Total phenols mg/g plant extract (in GAE)**	b **Total flavonoids mg/g plant extract (in QA)**	c **Total antioxidant capacity mg/g plant extract (in ASC)**
**MPBL**	39.92	136.33 ± 1.02*	52.16 ± 0.61*	81.72 ± 0.48*

The GAE, QA, and ASC values are expressed as Means±SEM of triplicate experiments.

a Galic acid equivalents (GAE, mg/g of each extract) for the total phenolic content,

b Quercetin equivalent (QA, mg/g of each extract) for the total flavonoid content,

c Ascorbic acid equivalent (ASC, mg/g of each extract) for the total antioxidant capacity.

**Table 2:** Scavenging/inhibitory effects of the *Piper betle* extract against DPPH, ONOO^-^, and Total ROS generation

**Sample**	**DPPH** **IC**_50_ **(µg/ml)**	**ONOO** ^-^ **IC**_50_ **(µg/ml)**	**ROS** **IC**_50_ **(µg/ml)**
**MPBL**	16.33±1.02*	25.16±0.61^*^	41.72±0.48*
**Ascorbic acid**	12.10±0.02		
**L-penicillamine**		10.20±0.32	
**Trolox**			12.32±0.19

IC_50_ values are mean±SEM (n=3);

* p < 0.001 by student t-test for values between the sample and the control.

***In vivo*** **analgesic activity**

*Hot plate method*

**Figure 2:**
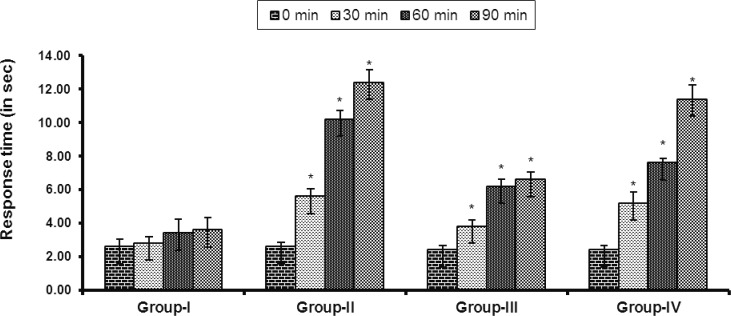
Effects of the MPBL on latency to hot plate test. Values are mean±SEM, (n=5); *p<0.05 as compared with vehicle control (one-way ANOVA followed by Dunnett’s test). Group I animals received vehicle (1% Tween 80 in water), Group II received nalbuphine 10 mg/kg body weight, Group III and Group IV were treated with 100 and 200 mg/kg body weight (p.o.) of the crude extract of *P. betle*, respectively

Result of hot plate test is shown in Figure 2. Both doses of the extract produced a dose-dependent increase in latency time when compared with the vehicle. The result was found to be statistically significant (p<0.05).

*Acetic acid-induced writhing test*

Table 3 shows the effects of the extract of on acetic acid-induced writhing in mice. The oral administration of both doses of MPBL significantly (p<0.05) inhibited writhing response induced by acetic acid in a dose-dependent manner.

*Formalin test*

MPBL (100 and 200 mg/kg, p.o.) significantly (P<0.05) suppressed the licking activity in either phase of the formalin-induced pain in mice in a dose-dependant manner (Table 4). MPBL, at the dose of 200 mg/kg body weight, showed almost similar licking activity against both phases of formalin-induced pain than that of the standard drug diclofenac Na.

**Anti-inflammatory activity**

*Carrageenan-induced paw edema test*

**Figure 3:**
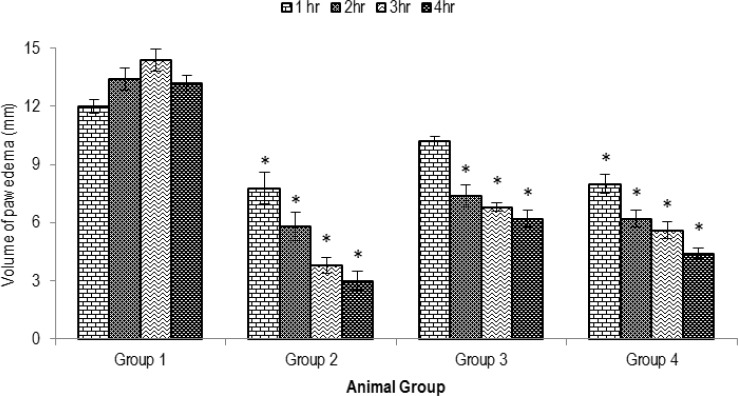
Effects of the MPBL on carrageenan-induced paw edema test. Values are mean±SEM, (n=5); *p<0.05 as compared to vehicle control (one-way ANOVA followed by Dunnett’s test). Group I animals received vehicle (1% Tween 80 in water), Group II received indomethacin10 mg/kg body weight, Group III and Group IV were treated with 100 and 200 mg/kg body weight (p.o.) of the crude extract of *P. betle*, respectively

Figure 3 shows the results of the anti-edematous effects of orally administered methanolic extract of *P. betle* on carrageenan-induced paw edema in rats. MPBL showed statistically significant (p<0.05) dose-dependent anti-inflammatory activity. MPBL showed remarkable anti-inflammatory effects at 200 mg/kg dose (66.66% inhibition), whereas standard indomethacin showed 72.72% of inhibition of paw edema. 

**Table 3:** Effects of the MPBL on acetic acid-induced writhing in mice

**Groups**	**Dose (mg/kg)**	**No. of writhing**	**% inhibition**
**Group I**	Vehicle	34.40	
**Group II**	10	10.60	69.19*
**Group III**	100	19.80	42.44*
**Group IV**	200	12.27	64.53*

Values are mean±SEM, (n=5);

* p<0.05 as compared with vehicle control (one-way ANOVA followed by Dunnett’s test). Group I animals received vehicle (1% Tween 80 in water), Group II received diclofenac Na 10 mg/kg body weight, Group III and Group IV were treated with 100 and 200 mg/kg body weight (p.o.) of the MPBL.

**Table 4:** Effect of MPBL in hindpaw licking in the formalin test in mice

**Groups**	**Dose (mg/kg)**	**Early phase (Sec)**	**% protection**	**Late phase (Sec)**	**% protection**
**Group-I**	Vehicle	34.16 ± 1.38	-	47.0 ± 1.03	-
**Group-II**	10	16.83 ± 0.90*	50.73	19.83 ± 0.70*	57.80
**Group-III**	100	27.5 ± 0.76*	19.51	21.5 ± 0.95*	54.25
**Group-IV**	200	18.00 ± 0.65*	47.31	20.67 ± 1.46*	56.02

Values are mean±SEM, (n=5);

* p<0.05 as compared to vehicle control (one-way ANOVA followed by Dunnett’s test). Group I animals received vehicle (1% Tween 80 in water), Group II received diclofenac Na 10 mg/kg body weight, Group III and Group IV were treated with 100 and 200 mg/kg body weight (p.o.) of the MPBL, respectively.

## Discussion

The upshots of oxidative stress are serious and sometimes manifested by increased activities of enzymes involved in oxygen detoxification (Gupta et al., 2005 ▶). Therefore, the identification of new antioxidant may reduce the risk of various chronic diseases involved in free radicals. To determine the efficacy of natural antioxidants either as pure compounds or as plant extract, a great number of *in vitro* methods have been developed in which antioxidant compounds act by several mechanisms. The knowledge of total antioxidant activity can be useful in the analysis of changes in plasma antioxidant activity related to oxidative stress, or the understanding of structure–activity relationships of pure antioxidant species. The phosphomolybdenum method was based on the reduction of Mo(VI) to Mo(V) by the compounds having antioxidant property and is successfully used to quantify vitamin E in seeds (Prieto et al., 1999 ▶). 

DPPH is a stable free radical that accepts an electron or hydrogen radical to become a stable diamagnetic molecule (Lompo et al., 2007 ▶) and is usually used as a substrate to evaluate the antioxidant activity of a compound (Braca et al., 2001 ▶). Based on the data obtained from this study, DPPH radical scavenging activity of MPBL (IC_50_ 16.33±0.16 µg/ml) was similar to the standard ascorbic acid (IC_50_ 12.10±0.02 μg/ml). Moreover, it was revealed that MPBL did show the proton donating ability and could serve as free radical inhibitor or scavenger. A direct correlation between antioxidant capacity and reducing power of certain plant extracts has been reported (Nakayama et al., 1993 ▶). 

The reducing properties are generally associated with the presence of reductones, which have been shown to exert antioxidant action by breaking the free radical chain by donating a hydrogen atom (Tanaka et al., 1988 ▶). Because a substance may act as an antioxidant due to its ability to reduce ROS by donating hydrogen atom (Jayprakash et al., 2001 ▶), the ferric reducing property of plant extracts (Figure 1) implies that they are capable of donating hydrogen atom in a dose-dependent manner. Polyphenolic compounds, such as flavonoids, tannins, and phenolic acids, which are commonly found in plants, have been reported to have multiple biological effects, including antioxidant activity (Khanam et al., 2004 ▶). Phenolic compounds are understood to induce the cellular antioxidant system and increase approximately 50% cellular glutathione concentration. *P. betle *leaves are rich in phenol, polyphenol, and tannin (Kahkonen et al., 1999 ▶) and may be responsible for causing the paramount antioxidant effect which is supported by previous studies (Arambewela et al., 2005 ▶; Choudhary and Kale, 2002 ▶; Santhakumari et al., 2003 ▶; Dasgupta and De, 2004 ▶.). 

The hot plate method is commonly used for assessing central antinociceptive response involving higher brain functions and is a supraspinally organized response (Chapman et al., 1985 ▶; Elisabetsky et al., 1995 ▶). Narcotic analgesics inhibit both peripheral and central mechanism of pain, while nonsteroidal anti-inflammatory drugs inhibit only peripheral pain (Pal et al., 1999 ▶; Ahmed et al., 2006 ▶). As noted, nalbuphine, the reference narcotic analgesic drug (5 mg/kg, p.o.) exhibited significant and paramount analgesic effects in the hot plate (supra spinal) test, whereas, MPBL (100 and 200 mg/kg, p.o.) produced a statistically significant but lesser in degree antinociceptive response to that of nalbuphine suggesting that the plant extract may act as a narcotic analgesic.

On the other hand, acetic acid-induced writhing response is a sensitive procedure to evaluate peripherally acting analgesics and represents pain sensation by triggering localized inflammatory response. Such pain stimulus leads to the release of free arachidonic acid from the tissue phospholipid (Ribeiro et al., 2000 ▶). The response is thought to be mediated by peritoneal mast cells (Voilley, 2004 ▶), acid sensing ion channels (Hossain et al., 2006 ▶), and the prostaglandin pathways (Adzu et al., 2003 ▶). The organic acid has also been postulated to act indirectly by inducing the release of endogenous mediators, which stimulates the nociceptive neurons that are sensitive to NSAIDs and narcotics (Alam et al., 2012 ▶). It is well known that non-steroidal anti-inflammatory and analgesic drugs mitigate the inflammatory pain by inhibiting the formation of pain mediators at the peripheral target sites where prostaglandins and bradykinin are proposed to play a significant role in the pain process (Kim et al., 2004 ▶). Therefore, it is likely that MPBL might have exerted its peripheral antinociceptive action by interfering with the local reaction caused by the irritant or by inhibiting the synthesis, release, and/or antagonizing the action of pain mediators at the target sites. The above findings clearly demonstrated that both central and peripheral mechanisms are involved in the antinociceptive action of MPBL. Interestingly, compounds such as flavonoids, steroids, and triterpenes in part, have been shown to possess anti-inflammatory and analgesic activity as the claim made by Pritam et al. (2011) ▶. Based on the classes of compounds detected in MPBL extract, several mechanisms of action could be used to explain the observed activities of MPBL extract. 

The formalin model normally postulates the site and the mechanism of action of the analgesic. This biphasic model is represented by neurogenic (0-5 min) and inflammatory pain (15-30 min), respectively (Hunskaar and Hole, 1987 ▶). Drugs that act primarily on the central nervous system such as narcotics inhibit both as steroids and NSAIDs suppress mainly the late phase (Alam et al., 2012 ▶). The suppression of neurogenic and inflammatory pains by the extract might imply that it contains active analgesic principle that may be acting both centrally and peripherally. This is an indication that the extract can be used to manage acute as well as chronic pain. The mechanism by which formalin triggers C-fibers activation remained unknown for a relatively long time. Recently, however, McNamara et al. (2007) ▶ demonstrated that formalin activates primary afferent neurons through a specific and direct effect on TRPA1, a member of the transient receptor potential family of cation channels, expressed by a subset of C-fiber nociceptors and this effect is accompanied by increased influx of Ca^2+^ ions. TRPA1 cation channels at primary sensory terminals were also reported to mediate noxious mechanical stimuli (Kerstein et al., 2009 ▶). These experiments suggest that Ca^2+^ mobilization through TRPA1 cation channels is concomitant with noxious chemicals and mechanical stimuli as they produce their analgesic action. It is likely that the inhibitory effect of MPBL to pain response is due to inhibiting the increase of the intracellular Ca^2+^ through TRPA1, presumably evoked by formalin. Therefore, MPBL may contain substances that affect the metabolism of Ca^2+^. 

Carrageenan-induced edema has been commonly used as an experimental animal model for acute inflammation and is believed to be biphasic. The early phase (1-2 h) of the carrageenan model is mainly mediated by histamine, serotonin, and increased synthesis of prostaglandins in the damaged tissue surroundings. The late phase is sustained by prostaglandin release and mediated by bradykinin, leukotrienes, polymorphonuclear cells, and prostaglandins produced by tissue macrophages (Antonio and Brito, 1998 ▶; Gupta et al., 2006 ▶; Sawadogo et al., 2006 ▶). Since the extract significantly inhibited paw edema induced by carrageenan in the second phase, this finding suggests a possible inhibition of cyclooxygenase synthesis by the extract and this effect is similar to that produced by non-steroidal anti-inflammatory drugs such as indomethacin, whose mechanism of action is inhibition of the cyclooxygenase enzyme. Flavonoids and saponins are well known for their ability to inhibit pain perception as well as anti-inflammatory properties due to their inhibitory effects on enzymes involved in the production of the chemical mediator of inflammation (Pin et al., 2010 ▶). This hypothesis is strongly supported by the previous study, which has shown that *P. betle *possess anti-inflammatory activity due to the presence of high flavonoid content (Koblyakov, 2001 ▶; Vaghasiya et al., 2007 ▶). 

In addition, the release of ROS and excessive nitric oxide (NO) due to the activation of neutrophils during tissue damage and inflammation is responsible for a variety of disease (Bhandare et al., 2010 ▶). Recent findings (Srivastava et al., 2000 ▶; Viana et al., 2003 ▶) suggest that polyphenols are potent inhibitors of NO synthase activity and NO production. As MPBL showed significant free radical as well as ONOO^-^ scavenging activity, this can be responsible for the reduction of inflammation in the carrageenan-induced paw edema in rats. The results of the experiments suggest that *P. betle *may be used as an alternative or supplementary herbal remedy for the treatment of pain and inflammatory disease. Because of its analgesic and anti-inflammatory effects, *P. betle* methanolic extract may have beneficial effects together with drugs known for having a strong analgesic as well as anti-inflammatory effects. Thus, the present study warrants further investigation involving components of *P. betle *for possible development of new class of analgesic and anti-inflammatory drugs. 
